# Activation of microglial GLP-1R in the trigeminal nucleus caudalis suppresses central sensitization of chronic migraine after recurrent nitroglycerin stimulation

**DOI:** 10.1186/s10194-021-01302-x

**Published:** 2021-07-29

**Authors:** Feng Jing, Qian Zou, Yangyang Wang, Zhiyou Cai, Yong Tang

**Affiliations:** 1 Department of Histology and Embryology, Chongqing Medical University, No.1 Yixueyuan Road, Yuzhong District, 400016 Chongqing, China; 2 Department of Neurology, Chongqing General Hospital, University of Chinese Academy of Sciences, No.118 Xingguang Avenue, Liangjiang New Area, 401147 Chongqing, China; 3 Chongqing Key Laboratory of Neurodegenerative Diseases, No.312 Zhongshan First Road, Yuzhong District, 400013 Chongqing, China

**Keywords:** GLP-1 receptor, Chronic migraine, Microglia, Central sensitization, Trigeminal nucleus caudalis, Nitroglycerin

## Abstract

**Background:**

Central sensitization is considered a critical pathogenic mechanism of chronic migraine (CM). Activation of microglia in the trigeminal nucleus caudalis (TNC) contributes to this progression. Microglial glucagon-like peptide-1 receptor (GLP-1R) activation can alleviate pain; however, whether it is involved in the mechanism of CM has not been determined. Thus, this study aims to investigate the precise role of GLP-1R in the central sensitization of CM.

**Methods:**

Repeated nitroglycerin injection-treated mice were used as a CM animal model in the experiment. To identify the distribution and cell localization of GLP-1R in the TNC, we performed immunofluorescence staining. Changes in the expression of GLP-1R, Iba-1, PI3K and p-Akt in the TNC were examined by western blotting. To confirm the effect of GLP-1R and PI3K/Akt in CM, a GLP-1R selective agonist (liraglutide) and antagonist (exendin(9–39)) and a PI3K selective antagonist (LY294002) were administered. Mechanical hypersensitivity was measured through von Frey filaments. To investigate the role of GLP-1R in central sensitization, calcitonin gene-related peptide (CGRP) and c-fos were determined using western blotting and immunofluorescence. To determine the changes in microglial activation, IL-1β and TNF-α were examined by western blotting, and the number and morphology of microglia were measured by immunofluorescence. We also confirmed the effect of GLP-1R on microglial activation in lipopolysaccharide-treated BV-2 microglia.

**Results:**

The protein expression of GLP-1R was increased in the TNC after nitroglycerin injection. GLP-1R was colocalized with microglia and astrocytes in the TNC and was fully expressed in BV-2 microglia. The GLP-1R agonist liraglutide alleviated basal allodynia and suppressed the upregulation of CGRP, c-fos and PI3K/p-Akt in the TNC. Similarly, the PI3K inhibitor LY294002 prevented nitroglycerin-induced hyperalgesia. In addition, activating GLP-1R reduced Iba-1, IL-1β and TNF-α release and inhibited TNC microglial number and morphological changes (process retraction) following nitroglycerin administration. *In vitro*, the protein levels of IL-1β and TNF-α in lipopolysaccharide-stimulated BV-2 microglia were also decreased by liraglutide.

**Conclusions:**

These findings suggest that microglial GLP-1R activation in the TNC may suppress the central sensitization of CM by regulating TNC microglial activation via the PI3K/Akt pathway.

**Supplementary Information:**

The online version contains supplementary material available at 10.1186/s10194-021-01302-x.

## Background

Chronic migraine (CM) is a severe neurological disease that seriously affects the daily life of patients and imposes a large economic burden on society [1, 2]. CM is the most common chronic headache in headache clinics in China [3]. The chronic condition of CM is attributable to repeated migraine attacks, and 2.5 % of episodic migraine converts into CM each year [4, 5]. Therefore, early prophylactic treatment is necessary for improving the prognosis of CM patients. However, the present treatments for CM are not satisfactory [6], and the pathogenesis of CM is still not fully understood.

Accumulating evidence indicates that central sensitization is a critical mechanism of CM, which manifests as neuronal plastic changes in the trigeminal nucleus caudalis (TNC) [7–9]. Studies have suggested that microglia may participate in crosstalk with neurons through chemotaxis, phagocytosis, proinflammatory cytokine and neurotrophin production [10–12]. In a cortical spreading depression (CSD) model [13, 14], which mimics the aura of migraine, and the chronic inflammatory soup (IS)-triggered CM model, microglia are dramatically activated [15, 16]. In addition, our previous studies revealed that microglia and their purinergic receptors (P2 × 4R, P2 × 7R and P2Y12R) were significantly upregulated in the TNC after NTG injection, and inhibiting microglial activation, including morphological and inflammatory changes, might affect neuronal hyperexcitability in the TNC, which ultimately relieved CM-associated allodynia [17–19]. These findings demonstrate that TNC microglia play a crucial role in the central sensitization of CM, and a better understanding of the function of TNC microglia will help us to further understand the pathogenesis of CM.

Recently, Wang Yongxiang and colleagues provided the first evidence that activation of microglial glucagon-like peptide-1 receptor (GLP-1R) in the spinal cord specifically suppresses neuropathic pain, cancer pain, and diabetic hypersensitivity [20]. However, whether GLP-1R is involved in the central sensitization of CM has not yet been studied. GLP-1 is an endogenous insulinotropic hormone secreted from L cells of the small intestine, that participates in the homeostatic regulation of insulin and glucose by activating GLP-1R [21, 22]. GLP-1R is a kind of G-protein-coupled receptor. Activation of GLP-1R can inhibit apoptosis and inflammation by stimulating phosphoinositide 3-kinase (PI3K) and protein kinase A (PKA) [23]. GLP-1R expressed in the central nervous system (CNS) [24] may also be involved in regulating cell proliferation, neuronal excitability and synaptic plasticity [25–27]. However, the distribution and function of GLP-1R in the TNC have not yet been examined. Previous studies have revealed that migraine patients suffer from impaired insulin sensitivity and have higher blood glucose levels [28]. In addition, insulin-like growth factor-1 (IGF-1) treatment can inhibit CSD and calcitonin-gene-related peptide (CGRP) production, thereby relieving migraine-related hyperalgesia [29]. These data combined with the effect of GLP-1R in chronic pain suggest that activation of GLP-1R may also alleviate CM.

Based on these results, we hypothesized that GLP-1R in the TNC mediates microglial activation via the PI3K/Akt pathway and suppresses the central sensitization of CM. In the present study, we investigated whether activation of GLP-1R prevented allodynia and inhibited microglial morphological changes and inflammation in the TNC using a chronic nitroglycerin (NTG) injection-stimulated mouse model and the selective GLP-1R agonist liraglutide. We also confirmed the role of GLP-1R on microglia in lipopolysaccharide (LPS)-incubated BV-2 cells. In addition, we examined the involvement of the PI3K/Akt pathway in CM. Our findings suggest for the first time that TNC microglial GLP-1R activation suppresses the central sensitization of CM and that GLP-1R may serve as a new target for treating CM.

## Materials and methods

### Animals

Male C57BL/6 mice weighing 18–20 g were kindly provided by the Experimental Animal Center of Chongqing Medical University (Chongqing, China). Animal experiments were conducted in accordance with Chongqing Medical University Animal Ethics Committee and were approved by the National Institutes of Health guidelines on animal care. Mice were housed with a 12 h light/dark cycle, at a stable temperature of 23 ± 2 °C and humidity of 50 ± 10 %, and were given food and water ad libitum. Every effort was made to minimize the number of mice used in the experiments and their suffering.

### Drug treatments

Nitroglycerin (NTG) (5.0 mg/ml, Henan Reagent, China) was freshly diluted to a final concentration of 1 mg/ml with saline, containing 6 % propylene glycol and 6 % alcohol. The intraperitoneal (i.p.) delivery of the diluted NTG was performed as described previously, that is, injected at a dose of 10 mg/kg every other day for 9 d (i.e., days 1, 3, 5, 7, and 9). Mice in the sham group were injected with an equivalent volume of saline, 6 % propylene glycol, and 6 % alcohol.

To measure the effect of GLP-1R in CM, a selective GLP-1R agonist (liraglutide) and an antagonist (exendin(9–39)) were applied in the experiment. Liraglutide (800 µg/kg; Selleck, TX, USA) and exendin(9–39) (50 µg/kg; MedChemExpress/MCE, USA), diluted with sterile saline, were administered i.p. for 16 consecutive days; that is, these drugs were administered 1 week before NTG injection. The time point for injections was also selected before NTG administration. To examine the role of PI3K/Akt in CM, the PI3K/Ak antagonist LY294002 (20 mg/kg; Selleck, TX, USA), diluted with sterile saline, was injected i.p. five times every other day prior to NTG injections. The dose and delivery method of these drugs were based on previous research [30–33] and our experience.

### Body weight and Blood glucose test

After a week of adaptation, the animals were randomly assigned to different experimental groups, as shown in Table 1 in which the experimental sets and sample size were delineated. During the whole experiment, the body weight and blood glucose of mice were measured 3 times, respectively, before drug administration, 1 week and 16 d after delivering liraglutide or exendin(9–39). The time point was selected at 9:00 prior to NTG injection. Glucose measurement was performed by a blood glucose meter (Sinocare, Changsha, China) using blood collected from the tail vein of mice.

**Table 1:** Schematic representation of experimental groups, sample size (number, n) and the sampling time (hour, h) of mice per group for different experimental analysis

Experimental groups	Total (n)	Behavioural test (n)	WB (other) (n)	WB (c-fos) (n)	IF (other) (n)	IF (c-fos) (n)	Sampling time (h)
Sham	9	-	6	-	3	-	24h after the first, the second, the third and the last NTG i.p. injections
NTG (1d)	6	-	6	-	-	-	
NTG (3d)	6	-	6	-	-	-	
NTG (5d)	6	-	6	-	-	-	
NTG (9d)	9	-	6	-	3	-	
Sham	20	8	6	6	4	4	For c-fos: 2h after the last NTG/saline i.p. injection; For other targets: 24h after the last NTG/saline i.p. injection
Exe(9-39)	12	8	6	6	-	-	
Lira	12	8	6	6	-	-	
CM	20	8	6	6	4	4	
CM+Exe(9-39)	20	8	6	6	4	4	
CM+Lira	20	8	6	6	4	4	
CM+LY294002	8	8	-	-	-	-	

WB (other), western blot for GLP-1, GLP-1R, CGRP, Iba-1, IL-1β, TNF-α, PI3K and p-AKT; IF (other): immunofluorescence for GLP-1R, CGRP, GFAP, NeuN and Iba-1; Exe(9-39), exendin(9-39); Lira, liraglutide

### BV-2 cell culture and treatment

BV-2 cells were provided by Procell Life Science & Technology (Wuhan, China). Cells were cultured in Dulbecco’s modified Eagle’s medium (DMEM) (Gibco, NY, USA) supplemented with 10 % foetal bovine serum (FBS) (Gibco, NY, USA) and were maintained in a humidified incubator at 37 °C and 5 % CO_2_.

Cells were incubated with serum-free media for 24 h prior to each experiment and seeded at 1 × 10^6^ cells/dish in 100 mm culture dishes for western blotting and 3 × 10^4^ cells/well in 24-well- culture plates for immunofluorescence. To study the role of GLP-1R in microglial inflammation, BV-2 cells were preincubated with the selective GLP-1R agonist liraglutide (100 nM, Selleck, TX, USA) and the antagonist exendin(9–39) (200 nM, MCE, USA) for 24 h, followed by LPS (1 µg/ml, Sigma–Aldrich, MO, USA) stimulation for 24 h. The doses of these drugs used with cells were based on previous data [34–36].

### Behavioural tests

Mice were acclimated to the testing chambers for 3 successive days before the behavioural tests. All experiments were performed in a low-light room between 9:00 and 15:00 at time points before and 2 h after NTG injection. The experimenter was blinded to the dug condition and the behavioural data analysis.

Both head-specific (periorbital) and hindpaw hyperalgesia were measured to reveal the hypersensitivity of the CM animal model. Mechanical responses were detected by a set of von Frey filaments (bending force ranging from 0.008 to 2 g, Aesthesio, USA) according to the up and down method [37, 38]. The first filament was chosed as 0.4 g. For periorbital testing, mice were placed in a paper cup to which they had previously been habituated, and then the periorbital region, positioned caudal to the eyes and near the midline, was stimulated with a von Frey filament. A positive response was considered quick retraction of the head or scratching of the face with the ipsilateral forepaw. In the absence of a response, a heavier filament (up) was applied, and in the presence of a response, a lighter filament (down) was applied. The duration of each stimulus was 3 s with an interval of at least 1 min. This process was followed for a maximum of 4 filaments after the first response. For hindpaw sensitivity, mice were habituated to acrylic cages 30 min before the test, and then the central area of the plantar surface of the hindpaw was stimulated with von Frey filaments. Positive responses were defined as lifting, shaking or licking of the paw. Testing was performed according to the up-down method described above.

### Western blotting

To analyze the protein expression of GLP-1, GLP-1R, PI3K and p-Akt on different days after NTG intermittent injection, TNC samples were collected 24 h after the first (NTG 1d), the second (NTG 3d), the third (NTG 5d) and the last (NTG 9d) NTG injections. To examine c-fos expression, TNC tissues were collected 2 h after the last NTG injection, while for other protein targets, TNC was isolated and collected 24 h after the last NTG administration. The sampling time was shown in Table 1. Tissues were homogenized in cold RIPA lysis buffer (Beyotime, Shanghai, China) containing phenylmethylsulfonyl fluoride (PMSF, Beyotime, Shanghai, China) at 4 °C for 1 h. The total amount of protein was determined using a BCA protein assay kit (Beyotime, Shanghai, China). The protein samples (40 µg) were separated by 10 % SDS-polyacrylamide gel (Beyotime, Shanghai, China) electrophoresis and transferred to PVDF membranes. Following transfer, the membranes were blocked in TBST containing 5 % skim milk for 2 h at room temperature (RT). Then, the blots were incubated with the following antibodies overnight at 4 °C: mouse anti-GLP-1R (1:200, Santa Cruz, CA, USA), mouse anti-GLP-1 (1:100, Santa Cruz, CA, USA), mouse anti-CGRP (1:100, Santa Cruz, CA, USA), mouse anti-c-fos (1:200, Santa Cruz, CA, USA), rabbit anti-Iba-1 (1:1000, Abcam, Cambridge, MA, USA), rabbit anti-PI3K (1:1000, Proteintech, China), rabbit anti-Akt (1:1000, Proteintech, China), rabbit anti-phospho-Akt (1:2000, Cell Signalling, Boston, MA, USA), rabbit anti-IL-1β (1:800, Wanleibio, China), rabbit anti-TNF-α (1:800, Wanleibio, China), and mouse anti-β-actin (1:1000, Beyotime, China). After the membranes had been washed with TBST, the secondary antibodies (goat anti-rabbit, 1:1000; goat anti-mouse, 1:1000; Beyotime, China) were applied and incubated for 1 h at RT. After the secondary antibody reaction, the bands were visualized with enhanced chemiluminescence, and the positive pixel area was detected by an image analysis system (Fusion, Germany). The band sensitivities were normalized against the corresponding β-actin loading controls. The western blotting test was repeated six times for each target, and consistent results were obtained.

To investigate the expression level of various proteins in BV-2 cells, we collected cells after LPS incubation. Cells were lysed in cold RIPA buffer containing PMSF for 30 min at 4 °C, and the total protein concentration was determined using a BCA protein assay kit. The following procedures were performed according to the method described above. Each analysis was repeated in three independent experiments.

### Immunofluorescence staining and counting

To stained c-fos, TNC tissues were collected 2 h after the last NTG injection, while for staining other targets, TNC was collected 24 h after the last NTG administration. Mice were deeply anaesthetized and perfused transcardially with 30 ml of PBS (pH 7.4) followed by 30 ml of cold 4 % paraformaldehyde (PFA) in PBS. The whole brain and cervical spinal cord (C1-C2) were collected and postfixed with 4 % PFA overnight at 4 °C.The medullary segment including the TNC between + 1 and − 3 mm from the obex was removed and transferred to 30 % sucrose for 48 h. After being frozen at − 80 °C, samples were cut into 10-µm-thick sections on a cryostat (Thermo). Sample sections were blocked in 5 % goat serum with 0.3 % Triton X-100 for 1 h at RT and then incubated overnight at 4 °C with the following primary antibodies: mouse anti-GLP-1R (1:50, Santa Cruz, CA, USA), mouse anti-GLP-1(1:50, Santa Cruz, CA, USA), rabbit anti-Iba1 (1:500, Wako Chemicals, Tokyo, Japan), rabbit anti-GFAP (1:200, Cell Signalling, Boston, MA, USA), rabbit anti-NeuN (1:100, Proteintech, China), mouse anti-CGRP (1:100, Santa Cruz, CA, USA), and mouse anti-c-fos (1:100, Santa Cruz, CA, USA). The sections were then incubated with fluorescence-conjugated secondary antibodies (Alexa Fluor Cy3 and Alexa Fluor 488, 1:500, Beyotime, China). Nuclei were stained with 4′,6-diamidino-2-phenylindole (DAPI) (Beyotime, China) at RT for 10 min. The sections were visualized and photographed with a confocal microscope (TCS Sp8, Leica). Quantification of the fluorescent signal intensity was performed by image analysis software (Image-Pro Plus 6.2, Media Cybernetics).

To quantify the immunoreactivity of CGRP and the numbers of c-fos- and iba-1- positive cells in the TNC, 6 sections per mouse from 4 mice were selected randomly for each group, and 6–8 visual fields per section were captured. Images centred on the superficial layer of the TNC were captured under a ×100 or × 200 objective, and the immunoreactive cells in this region were counted with Image-Pro Plus.

To quantify the length of microglial processes, 6–8 visual fields per section were selected from 6 sections per mouse (*n* = 4), and all intact cells in the field of view were measured. Skeletal images of each cell were drawn by Neuron J (an ImageJ plug-in), and then the total and mean lengths of microglial processes were automatically determined by the software.

### Statistical analysis

Statistical analyses were performed by SPSS 22.0 software (IBM Corp, Armonk, NY, USA). All data are expressed as mean ± SEM. The statistical significance of changes between the values was determined by one-way ANOVA with a post hoc test (Tukey’s test). Behavioural data were evaluated by two-way ANOVA with a Bonferroni post hoc test. *P* < 0.05 was considered to be statistically significant.

## Results

### Upregulation of GLP-1R in the TNC after chronic NTG injection

**Fig. 1:**
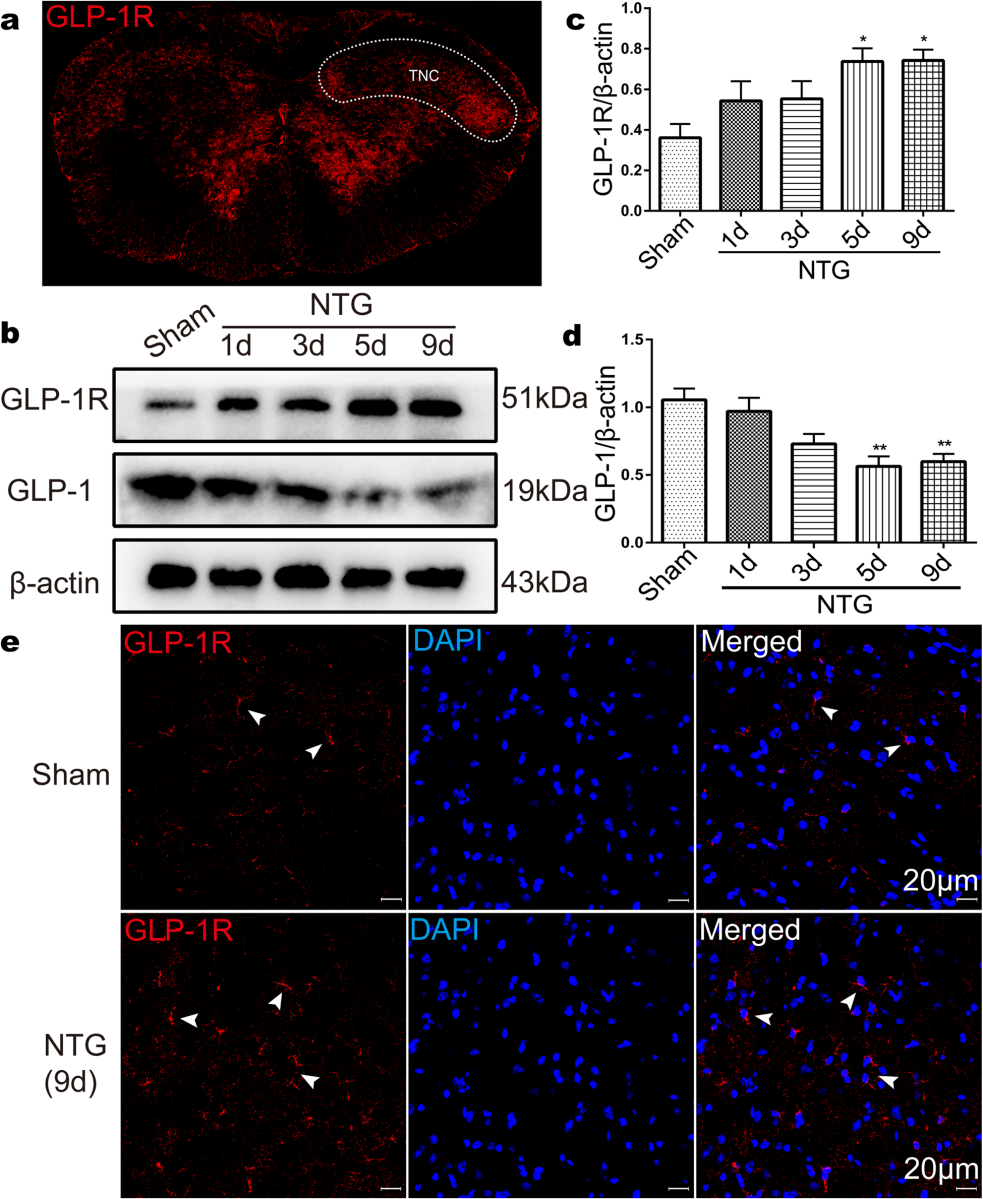
Recurrent NTG injection increased GLP-1R expression in the TNC. **a** The white dotted line frame exhibits the TNC regions. **b-d** Representative western blot showing changes in GLP-1R and GLP-1 protein expression in the TNC on different days after NTG injection (**b**). The band intensities are presented as the percent (%) change relative to β-actin (**c-d**). Data are presented as mean ± SEM; *n* = 6/group; *p < 0.05, **p < 0.01 vs. sham group. **e** Representative immunofluorescence images of GLP-1R in the TNC in the sham and NTG (9d) groups. Arrowheads indicate GLP-1R. *n* = 3/group, scale bar: 20 μm

To examine whether GLP-1R was expressed in the TNC, we performed immunofluorescence for GLP-1R using a specific antibody, and our images showed that GLP-1R was specifically expressed in the TNC in normal mice (Fig. 1a). To study the effect of NTG on GLP-1R activity, we used western blotting to detect the level of GLP-1R in the TNC and found that the expression of GLP-1R was increased after chronic NTG injection in a time-dependent manner (Fig. 1b) and peaked on day 9 (0.74 ± 0.13) (Fig. 1c, *p* < 0.05). In addition, we detected the protein level of GLP-1 in the TNC after NTG administration, which is a natural agonist of GLP-1R. We observed that recurrent NTG injection induced a time-dependent decrease in GLP-1 levels in the immunoblot analyses (Fig. 1b). GLP-1 expression was significantly decreased on day 5 (0.56 ± 0.18) and day 9 (0.60 ± 0.14) (Fig. 1d, *p* < 0.01) after NTG injection. We also performed immunofluorescence and observed that the number of GLP-1R-positive cells in the NTG (9d) group was higher than that in the sham group (Fig. 1e). These results indicate that GLP-1R is dramatically upregulated in the TNC in NTG-induced CM model.

### Localization of GLP-1R in the TNC

**Fig. 2:**
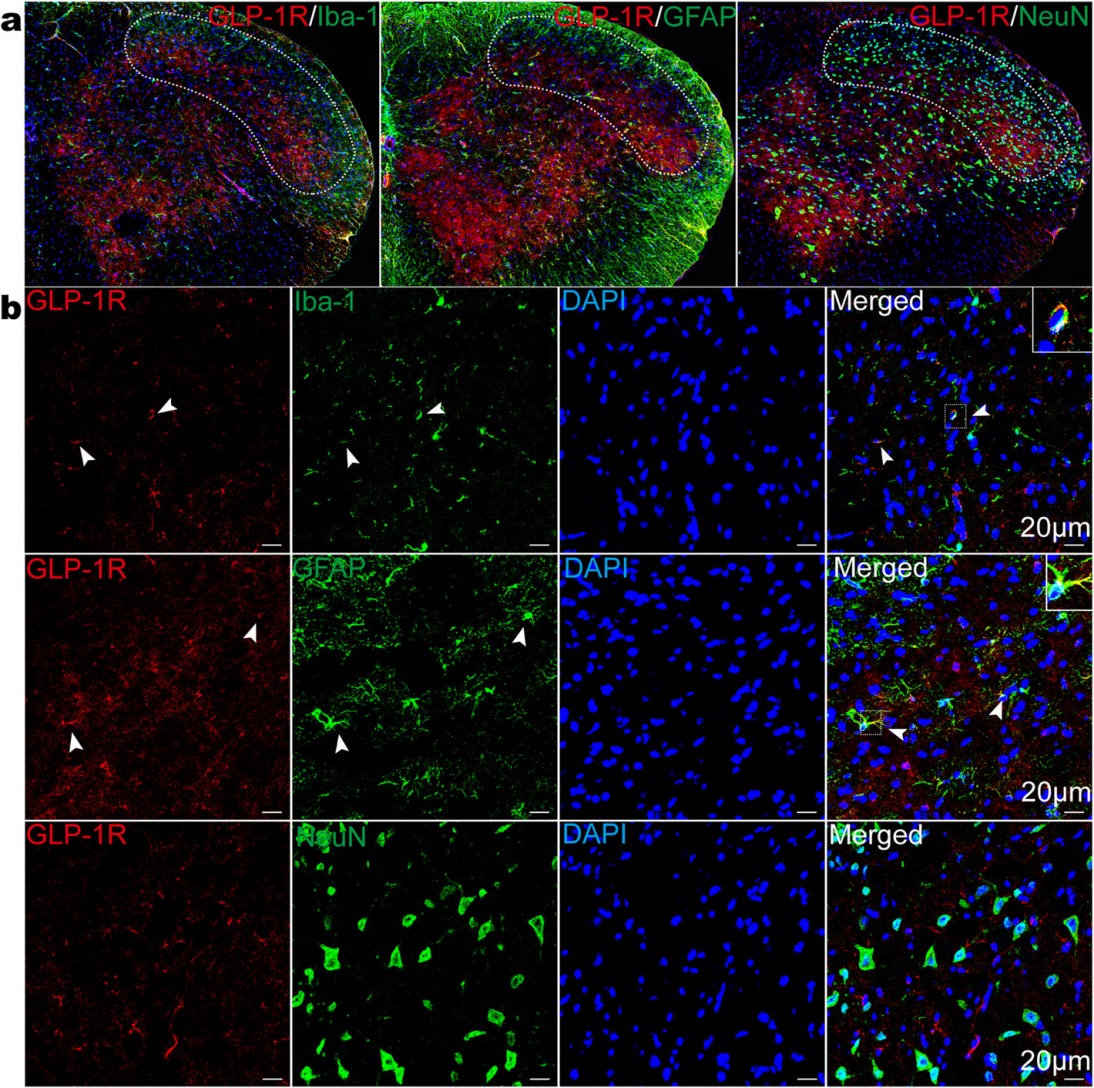
GLP-1R immunoreactivity localized to microglia and astrocytes in the TNC. **a, b** Double immunofluorescence labelling of GLP-1R (red) with Iba1, GFAP and NeuN (green) in the TNC in CM mice after NTG injection. The graphs in **a** show the TNC regions, which are marked by the white dotted line frame. The graphs in **b** show higher magnification images. Arrowheads show double-labelled cells. Scale bar: 20 μm

To localize the specific cell type for GLP-1R in the TNC, immunofluorescence was stained with Ibal-1 (microglial marker), GFAP (astrocytic marker) and NeuN (neuronal marker) using TNC sections in the CM group (Fig. 2a). Our images showed that GLP-1R-positive cells were mainly double labelled with Iba-1 and GFAP but did not colocalize with NeuN (Fig. 2b). These results suggest that GLP-1Rs exclusively localize to glial cells but not to neuronal cells.

### Activation of GLP-1R attenuated CM-associated mechanical allodynia

**Fig. 3:**
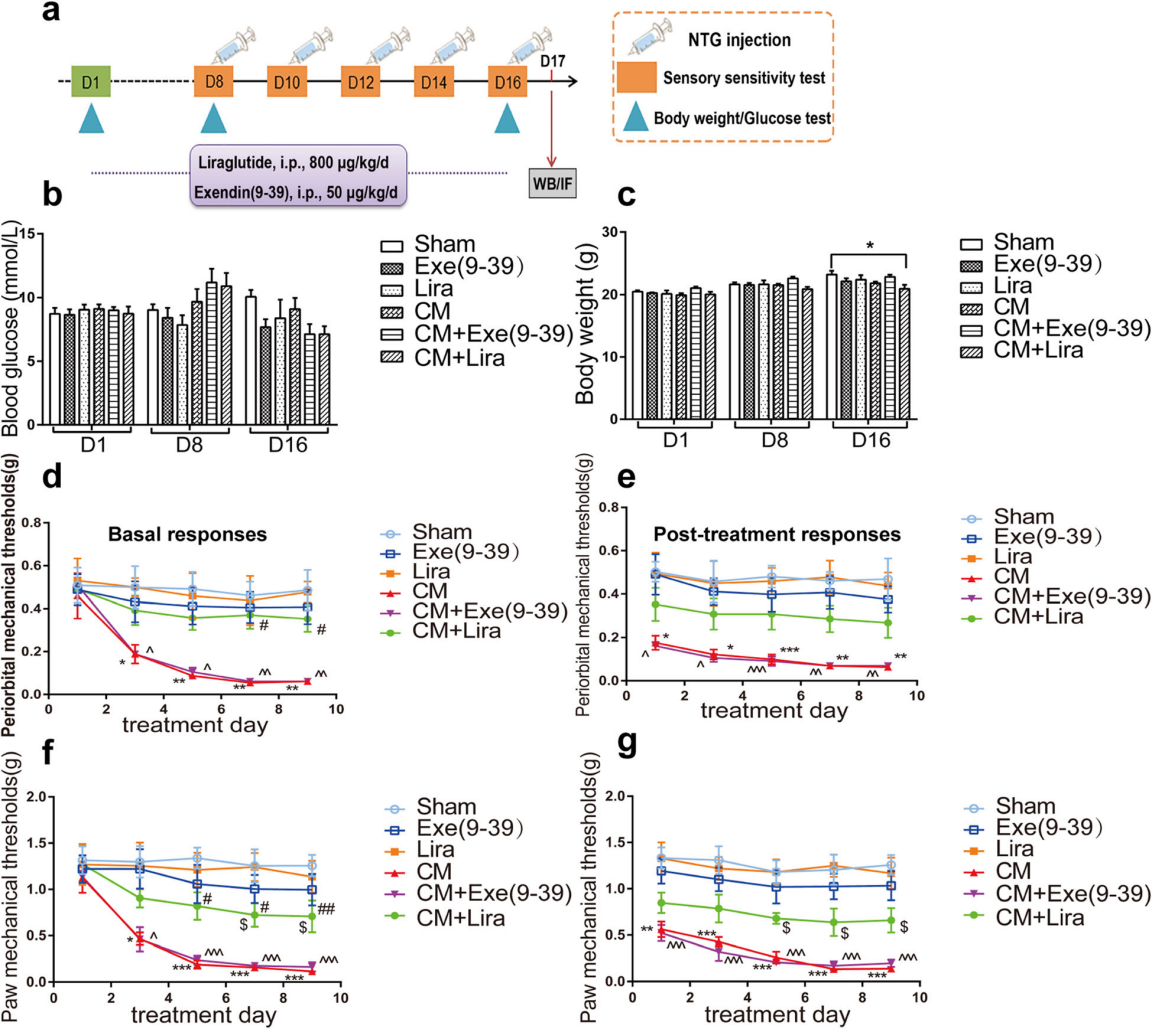
Effect of GLP-1R on the glucose level, body weight, and mechanical hyperalgesia of CM mice. **a** The schematic of experimental outline. **b, c** The graph shows the effect of liraglutide and exendin(9–39) on random blood glucose (**b**) and body weight (**c**). The results showed that chronic liraglutide treatment decreased the body weight of CM mice compared with that of the sham group. There were no differences between various groups in terms of glucose concentration. Data are presented as mean ± SEM; *n* = 8/group; *p < 0.05 vs. sham group. **d-g** Repeated daily injection of liraglutide prevented basal periorbital (**d**) and hindpaw (**f**) mechanical allodynia. Post-treatment responses, including periorbital (**e**) and hindpaw (**g**) mechanical hyperalgesia, were assessed 2 h after NTG administration. Liraglutide did not significantly attenuate NTG-triggered acute hyperalgesia. Data are presented as mean ± SEM; *n* = 8/group; two-way ANOVA and Bonferroni post hoc analysis; ^*,^,$^*p* < 0.05, ^**,^^^*p* < 0.01 and ^***,^^^^*p* < 0.001 CM, CM + Exe(9–39), CM + Lira groups, respectively, vs. sham group; ^#^*p* < 0.05 and ^##^*p* < 0.01 CM + Lira group vs. CM group. Exe(9–39), exendin(9–39); Lira, liraglutide

Considering the significant upregulation of GLP-1R in the TNC and the widely recognized effect of microglia in CM, it is reasonable to expect that GLP-1R may play an important role in the pathogenesis of CM. To study whether GLP-1R activation in TNC microglia can alleviate NTG-induced allodynia, the selective GLP-1R agonist liraglutide (800 µg/kg, *n* = 8 per group) and the antagonist exendin(9–39) (50 µg/kg, *n* = 8 per group) were i.p. injected for 16 d (Fig. 3a). The injection of the drugs started 1 week before NTG administration.

Liraglutide is a commonly used hypoglycaemic agent and is known to improve glucose homeostasis in diabetic patients [39]. To avoid the influence of hypoglycaemia on the experiment, we evaluated the changes in blood glucose and body weight of the experimental mice (Fig. 3b-c). The results showed that there was no significant difference in the levels of blood glucose in the various groups (Fig. 3b). Interestingly, the body weight of the CM combined with liraglutide group was significantly reduced compared with that of the sham group (Fig. 3c).

The periorbital and paw withdrawal thresholds were measured every other day from 1 to 9 d after NTG injection. The basal responses were evaluated before NTG, and the post-treatment responses were evaluated 2 h after NTG administration (Fig. 3d-g). We found that liraglutide (CM + Lira) markedly alleviated basal periorbital and paw hypersensitivity (*p* < 0.05). Chronic exendin(9–39) (CM + Exe(9–39)) injection did not improve CM-associated allodynia. Only liraglutide and exendin(9–39) injection did not provoke a significant effect on mechanical hypersensitivity.

### Activation of GLP-1R suppressed NTG-induced CGRP expression in the TNC

**Fig. 4:**
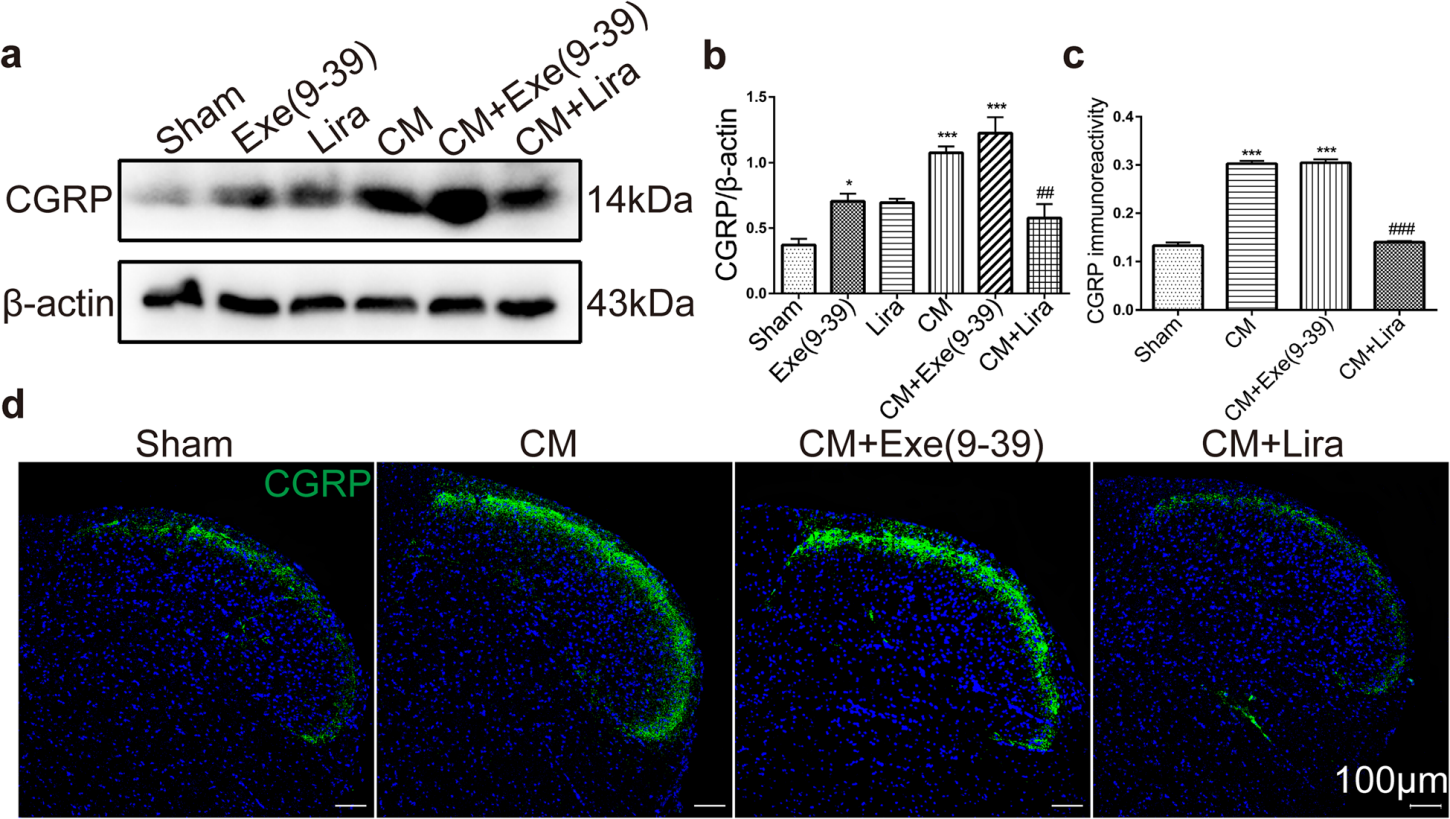
Activation of GLP-1R reduced the upregulation of CGRP expression in the TNC in CM mice. **a, b** Western blots showing the alteration of CGRP protein levels in the TNC treated with liraglutide and exendin(9–39). The band intensities are presented as the percent (%) change relative to the sham group. Values are presented as mean ± SEM; *n* = 6/group; *p < 0.05 and ***p < 0.001 vs. sham group; ^##^*p* < 0.01 vs. CM group. **c, d** Representative immunofluorescence staining images of CGRP in the TNC (**c**) and the quantitative analysis of CGRP immunoreactivity (**d**). The results showed that liraglutide inhibited NTG-induced CGRP immunoreactivity, which was consistent with the western blot assessment. Data are presented as mean ± SEM; *n* = 4/group; six sections from each mouse; ***p < 0.001 vs. sham group; ^###^*p* < 0.001 vs. CM group. Scale bars: 100 μm

CGRP is a widely recognized migraine-related mediator and is also involved in central sensitization in the TNC. To further confirm the effect of GLP-1R on central sensitization of CM, we measured alterations in CGRP expression in different drug-treated groups. The western blotting results showed that the protein level of CGRP in the TNC was significantly increased in the CM (*p* < 0.001) and GLP-1R antagonist exendin(9–39) treated groups (*p* < 0.05, Exe(9–39) vs. sham; *p* < 0.001, CM + Exe(9–39) vs. sham) (Fig. 4a-b). Activation of GLP-1R by its agonist liraglutide (CM + Lira) markedly suppressed the expression of CGRP compared with the corresponding level in the CM group (Fig. 4b, 0.58 ± 0.26 vs. 1.08 ± 0.12, *p* < 0.01). In addition, we performed immunofluorescence assessment of CGRP in the TNC (Fig. 4d). The density of CGRP-immunoreactive fibres in the TNC was increased in the CM and exendin(9–39) treated (CM + Exe(9–39)) groups compared with the sham group (Fig. 4c, *p* < 0.001). Consistent with the changes in CGRP levels in immunoblot analyses, the immunoreactivity of CGRP in the TNC in the CM group was significantly decreased by liraglutide treatment (Fig. 4c, *p* < 0.001).

### Activation of GLP-1R reduced NTG-induced c-fos expression in the TNC

**Fig. 5:**
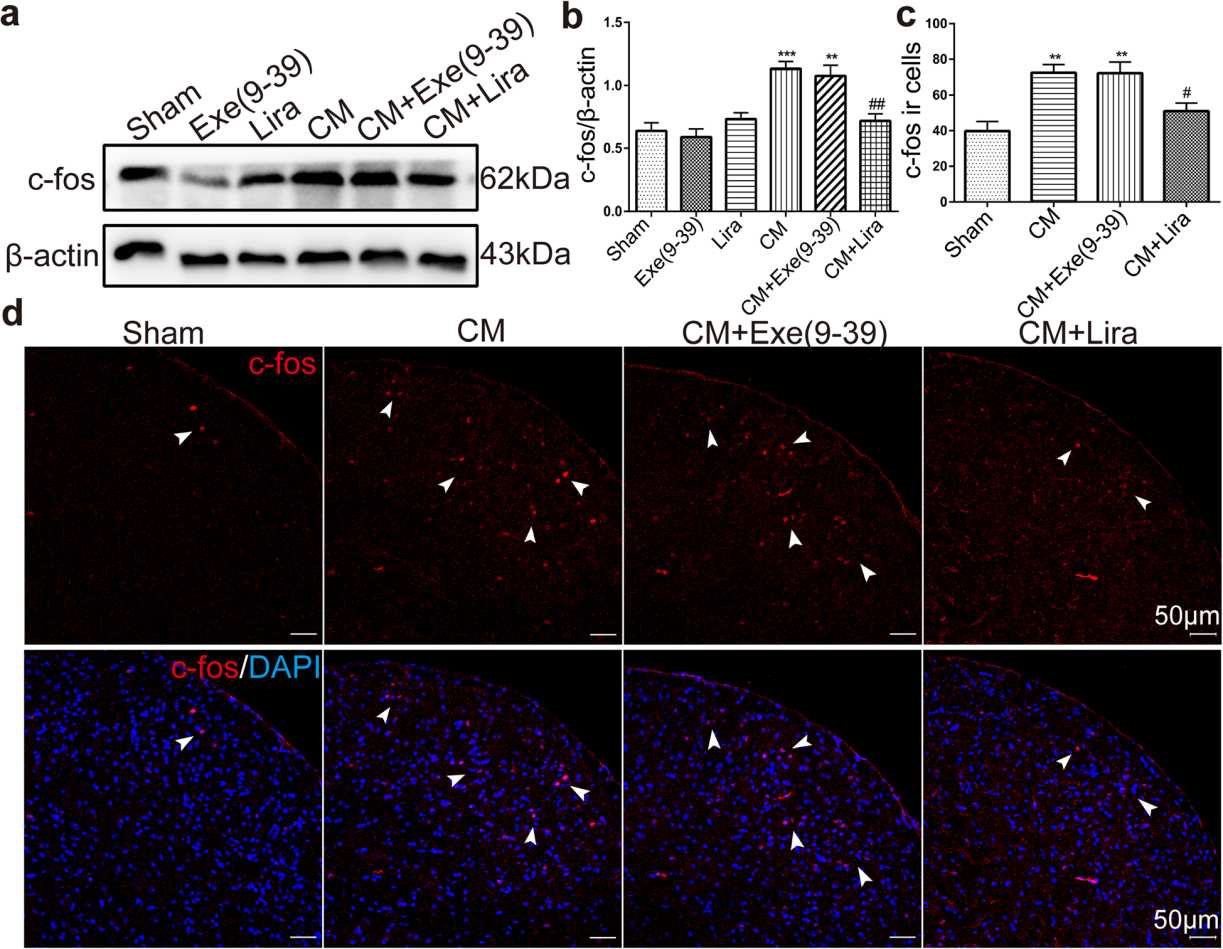
Activation of GLP-1R reduced the c-fos protein level and the immunoreactive cells in the TNC in CM mice. **a, b** Western blots showing the changes in c-fos protein expression in the TNC following liraglutide and exendin(9–39) administration. The band intensities are presented as the percent (%) change relative to the sham group. Data are presented as mean ± SEM; *n* = 6/group; **p < 0.01 and ***p < 0.001 vs. sham group; ^##^*p* < 0.01 vs. CM group. **c, d** Representative immunofluorescence images of c-fos in the TNC and the quantitative analysis of c-fos immunoreactive (ir) cells. Arrowheads indicate colocalization of c-fos-ir cells and DAPI. The results suggested that liraglutide reduced c-fos-ir cells in the TNC in CM mice. Data are presented as mean ± SEM; *n* = 4/group; six sections from each mouse; **p < 0.01 vs. sham group; ^#^*p* < 0.05 vs. CM group. Scale bars: 50 μm

In addition to CGRP, we also detected alterations in c-fos expression in the TNC, which is a reliable marker of neuronal activation and is considered another mediator of central sensitization. Using western blotting, we found that the protein levels of c-fos in the TNC in the CM (1.13 ± 0.14) and exendin(9–39) treated group (1.08 ± 0.20) were significantly upregulated (Fig. 5a-b, *p* < 0.001 CM vs. Sham; *p* < 0.01 CM + Exe(9–39) vs. Sham), and liraglutide treatment markedly suppressed the expression of c-fos in CM mice (Fig. 5a-b, 0.72 ± 0.14, *p* < 0.01 CM + Lira vs. Sham). Consistent with the changes of c-fos levels in immunoblot analyses, the immunofluorescence results for c-fos in the TNC showed that the number of c-fos immunoreactive cells in the exendin(9–39) treated group (CM + Exe(9–39), 72.33 ± 14.95) was higher than that of sham group (39.83 ± 13.23) (Fig. 5c-d, *p* < 0.01); however, the number of c-fos- positive cells in the liraglutide treated group (CM + Lira, 51 ± 11.22) was significantly decreased compared with that in the CM group (72.67 ± 10.69) (Fig. 5c-d, *p* < 0.05).

### GLP-1R contributes to microglial activation in the TNC in NTG-induced CM mice

**Fig. 6:**
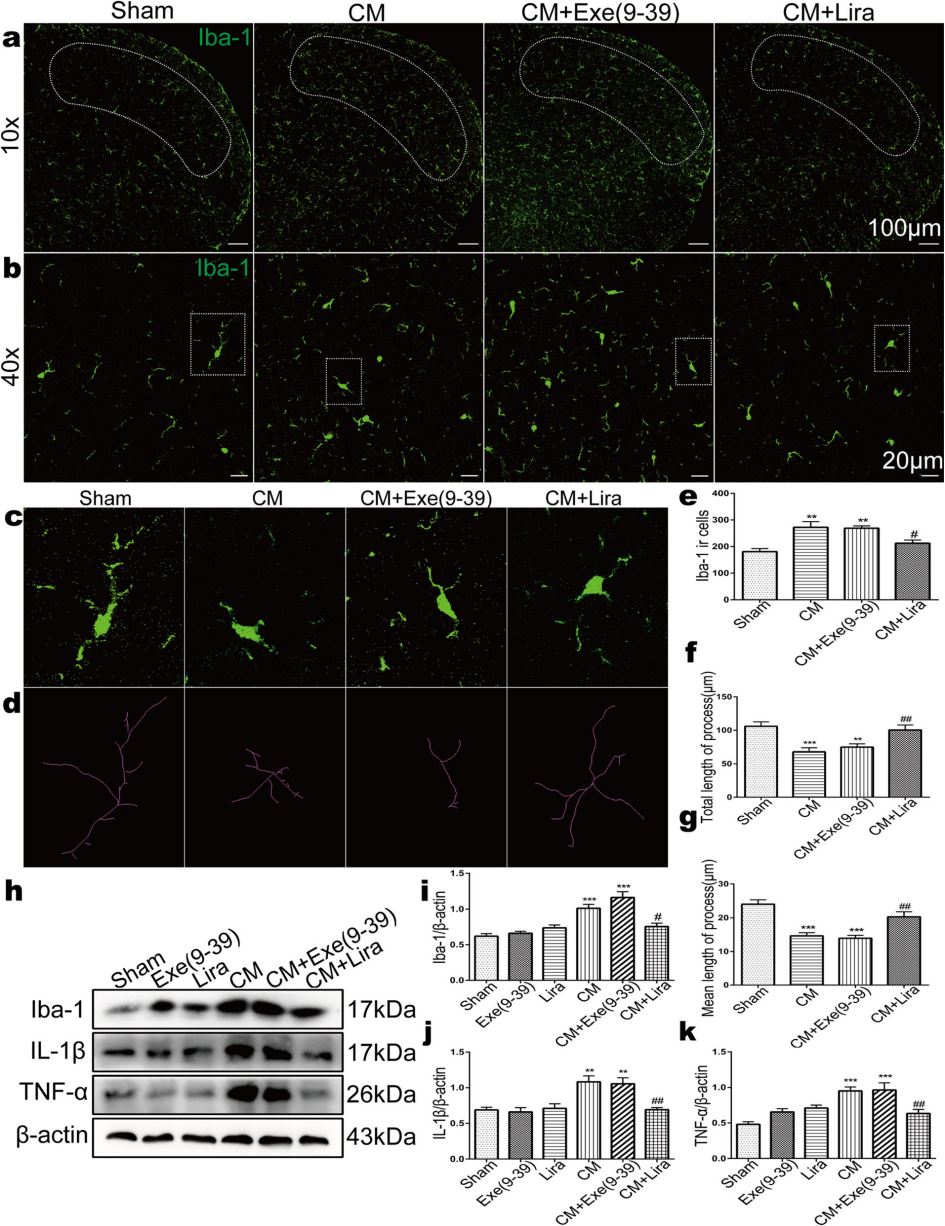
GLP-1R affected NTG-stimulated microglial morphological activation and proinflammatory cytokine release in the TNC. **a, b** Representative immunofluorescence images of Iba-1 in the TNC in the sham, CM, CM + Exe (9–39), and CM + Lira groups under an x 10 objective lens (**a**) and x 40 objective lens (**b**). Scale bar: 100 μm for x 10 images, 20 μm for x 40 images. **c** Magnified images of Iba-1 in the region enclosed in the white dotted line frame in **b**. **d** The transformed skeletal images of microglia in **c**. **e** Quantification of Iba-1-positive cells showed that liraglutide reduced the number of TNC microglia in CM mice. **f, g** Quantitative analysis of the total length (**f**) and mean length (**g**) of microglial processes in the TNC. The results showed that liraglutide treatment partially reversed the NTG-induced shortening of microglial processes in the TNC. Data are presented as mean ± SEM; *n* = 4/group; six sections from each mouse; **p < 0.01, ***p < 0.001 vs. sham group. ^#^*p* < 0.05 and ^##^*p* < 0.01 vs. CM group. **h-k** Western blots showing the changes in Iba-1, IL-1β and TNF-α protein levels in the TNC following liraglutide and exendin(9–39) treatment. The band intensities are presented as the percent (%) change relative to the sham group. Values are presented as mean ± SEM; *n* = 6/group; **p < 0.01 and ***p < 0.001 vs. sham group; ^#^*p* < 0.05, ^##^*p* < 0.01 vs. CM group

We have indicated that chronic NTG stimulation induces an increase in the number and activity of microglia in the TNC. To study the involvement of GLP-1R in these changes, we measured microglia from TNC slices of different drug-treated groups using immunofluorescence. Quantification of Iba1-positive cells revealed that the number of microglia in the CM (272.6 ± 47.44) and exendin(9–39) treated group (CM + Exe (9–39), 269.2 ± 19.04) was significantly increased compared with that in the sham group (180.8 ± 26.41) (Fig. 6a,e, *p* < 0.01). The agonist liraglutide markedly suppressed the NTG-induced upregulation of microglia in the TNC (Fig. 6a, e, CM + Lira 212.6 ± 26.92, *p* < 0.05). We observed that the activated microglia in the TNC in CM mice manifested as hypertrophic somata and shortened processes; thus, we quantified the microglial processes in the highly magnified stacked images of Iba1-immunoreactive cells in the TNC (Fig. 6b-c). In addition, skeletal images of microglia in various groups were drawn for accurate quantification (Fig. 6d). The results showed that GLP-1R agonist liraglutide treatment partially reversed the NTG-induced shortening of both total (100.45 ± 40.55 μm vs. 67.99 ± 31.03 μm, *p* < 0.01) and mean lengths (20.28 ± 8.11 μm vs. 14.63 ± 5.20 μm, *p* < 0.01) of microglial processes in the TNC (Fig. 6f-g).

In addition, we examined the protein level of Iba-1 in the TNC using western blotting and found that liraglutide markedly reduced NTG-induced upregulation of Iba-1 (CM + Lira 0.76 ± 0.12 vs. CM 1.01 ± 0.14, *p* < 0.05) (Fig. 6h-i). We also detected the expression of the microglial proinflammatory factors IL-1β and TNF-α in the TNC during chronic NTG administration. The results showed that the protein levels of both IL-1β (*p* < 0.01) and TNF-α (*p* < 0.001) were increased after recurrent NTG and exendin(9–39) injection (Fig. 6h, j-k). However, the release of IL-1β and TNF-α in the TNC in CM mice was significantly reversed by chronic liraglutide treatment (Fig. 6h, j-k, *p* < 0.01). Together, these results indicate that GLP-1R activity contributes to alterations in the number, morphology and inflammatory factor production of microglia in the TNC following NTG administration.

### Effect of GLP-1R on LPS-stimulated BV-2 microglial inflammatory cytokine release

**Fig. 7:**
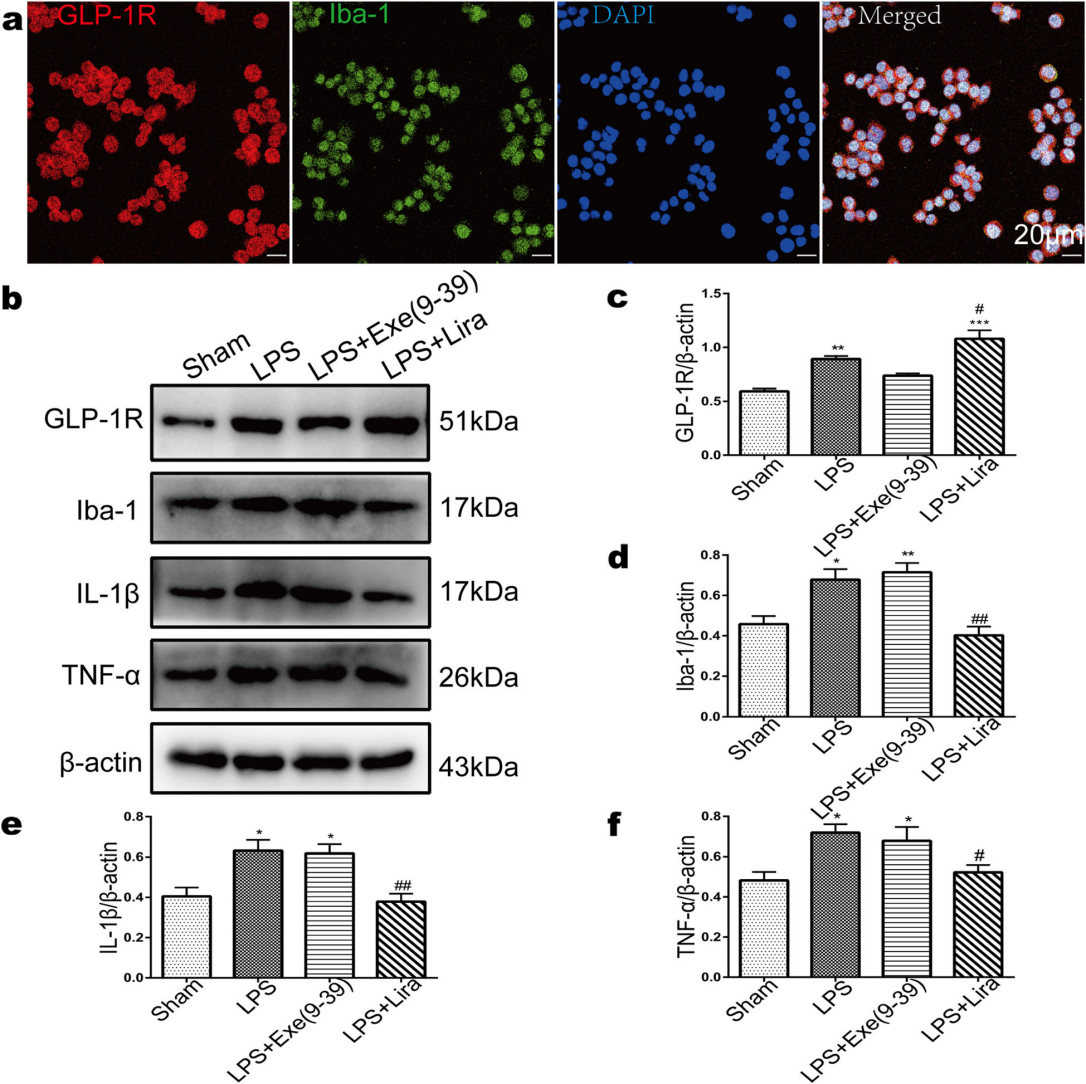
Activation of GLP-1R inhibited LPS-stimulated BV-2 microglial inflammatory cytokine release. **a** Double immunofluorescence staining images of GLP-1R with Iba-1 in BV-2 cells. Scale bar: 20 μm. **b** Western blots showing the alterations of GLP-1R, Iba-1, IL-1β and TNF-α protein levels in LPS-triggered BV-2 cells following liraglutide and exendin(9–39) incubation. **c-f** The graphs show the quantification of band intensities, which are presented relative to those of β-actin. Data are presented as mean ± SEM of three independent experiments; *p < 0.05, **p < 0.01 and ***p < 0.001 vs. sham group; ^#^*p* < 0.05, ^##^*p* < 0.01 vs. LPS group

Because liraglutide and exendin(9–39) were administered systematically *in vivo*, to further determine the effect of GLP-1R on microglial function, we carried out experiments in BV-2 microglia. Using double-labelling immunofluorescence, we observed that GLP-1R was colocalized with Iba1 in BV-2 cells (Fig. 7a). After LPS stimulation, the expression of GLP-1R was significantly increased compared with that of the sham group (Fig. 7b-c, 0.89 ± 0.06 vs. 0.59 ± 0.06, *p* < 0.01). Exendin(9–39) incubation partially reversed the expression of GLP-1R induced by LPS. However, the agonist liraglutide induced higher upregulation of GLP-1R compared with that in the LPS group. (Fig. 7b-c, 1.08 ± 0.19 vs. 0.89 ± 0.06, *p* < 0.05). We also quantified the expression of Iba-1, IL-1β and TNF-α after drug treatment, and the results showed that liraglutide reduced the protein level of Iba-1 (0.40 ± 0.11 vs. 0.68 ± 0.13, *p* < 0.01) and reversed the production of IL-1β (0.38 ± 0.09 vs. 0.63 ± 0.13, *p* < 0.01) and TNF-α (0.52 ± 0.09 vs. 0.72 ± 0.10, *p* < 0.05) induced by LPS (Fig. 7b, d-f). These results suggest that microglial GLP-1R mediates the inflammatory factor release of BV-2 microglia.

### GLP-1R is required for PI3K/Akt activation in the TNC following recurrent NTG injection

**Fig. 8:**
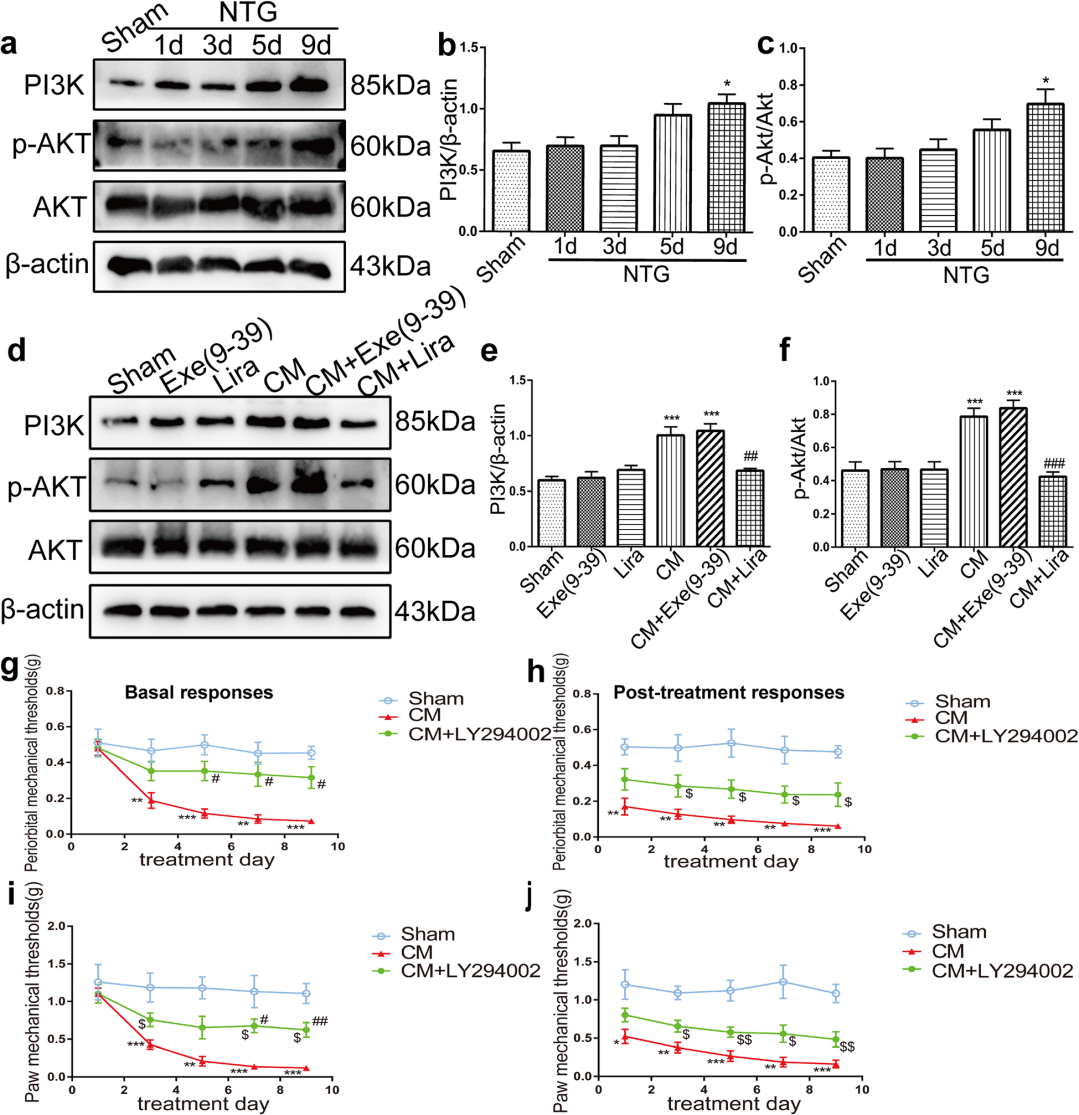
Inhibition of PI3K/Akt pathway activity, which is regulated by GLP-1R, prevented NTG-induced allodynia. **a-c** Representative immunoblots of PI3K and p-Akt (Ser473) in the TNC on different days following NTG injection. The band intensities of PI3K and p-Akt are presented relative to those of β-actin and total Akt, respectively. Quantitative analysis revealed a dramatic increase on day 9 after NTG administration. Data are presented as mean ± SEM; *n* = 6/group; *p < 0.05 vs. sham group. **d-f** Representative western blots showing the changes in PI3K and p-Akt (Ser473) protein expression in the TNC after liraglutide and exendin(9–39) treatment. The results showed that activation of GLP-1R by liraglutide markedly suppressed the increase in PI3K and p-Akt in the TNC in CM mice. Data are presented as mean ± SEM; *n* = 6/group; ***p < 0.001 vs. the sham group; ^##^*p* < 0.01, ^###^*p* < 0.001 vs. the CM group. Repeated treatment with the PI3K selective inhibitor LY294002 partially attenuated CM-associated basal periorbital (**g**) and hindpaw (**i**) mechanical hyperalgesia. Post-treatment responses, including periorbital (**h**) and hindpaw (**j**) mechanical allodynia, were assessed 2 h following NTG injection; however, LY294002 had no significant effect on NTG-induced acute allodynia. Data are presented as mean ± SEM; *n* = 8/group; two-way ANOVA and Bonferroni post hoc analysis; *^,$^*p* < 0.05, ^**,$$^*p* < 0.01 and ***p < 0.001 The CM and CM + LY294002 groups vs. the sham group; ^#^*p* < 0.05 and ^##^*p* < 0.01 vs. the CM group

Using western blotting, we measured the protein expression of PI3K and p-Akt in the TNC after repeated NTG injection (1, 3, 5, and 9 d post-injection) and found that these protein levels gradually increased in a time-dependent manner and peaked on day 9 (*p* < 0.05, PI3K in NTG 9d, 1.05 ± 0.18 vs. Sham 0.66 ± 0.17; *p* < 0.05, p-Akt in NTG 9d 0.69 ± 0.19 vs. Sham 0.40 ± 0.09) (Fig. 8a-c). Following recurrent treatment with the GLP-1R agonist liraglutide (CM + Lira), we observed a significant decrease in the expression of PI3K (0.68 ± 0.05 vs. 1 ± 0.18, *p* < 0.01) and p-Akt (0.42 ± 0.07 vs. 0.79 ± 0.12, *p* < 0.001) (Fig. 8d-f). The GLP-1R antagonist exendin(9–39) had no effect on NTG-induced PI3K and p-Akt expression levels. These results reveal that GLP-1R regulates PI3K/p-Akt activity in the TNC.

### Inhibition of PI3K/Akt activity alleviated NTG-induced mechanical hypersensitivity

To evaluate whether PI3K/ Akt is involved in NTG-induced pain hypersensitivity, we i.p. injected the PI3K antagonist LY294002 (20 mg/kg) 5 times prior to NTG injection every other day (*n* = 8 per group). The behavioural results showed that repeated LY294002 treatment partially reversed basal periorbital and paw hyperalgesia (Fig. 8g-j), which was markedly attenuated on days 7 and 9. However, LY294002 administration had no significant improvement in post-treatment allodynia induced by NTG. These data indicate that PI3K/Akt, downstream of GLP-1R, contributes to the development of CM.

## Discussion

In the current study, we observed the following new findings. (1) Chronic NTG injection induced a dramatic increase in GLP-1R in TNC microglia. (2) Activation of GLP-1R attenuated CM-associated allodynia and reduced the expression of CGRP and c-fos. (3) Activation of GLP-1R suppressed microglial morphological changes and inflammatory factor release in the TNC in CM mice and *in vitro*. (4) The PI3K/Akt pathway, inhibited by GLP-1R in the TNC, was proven to participate in the development of CM.

Previous studies have found that GLP-1Rs are expressed in various tissues, including the pancreatic islets, brain, dorsal root ganglia, and spinal cord [20, 40–43]. Regarding the cellular localization of GLP-1R in the CNS, there are controversial findings. In the cerebral cortex, GLP-1R is expressed on neurons and glia [42], while in the spinal cord, GLP-1R is coexpressed only with microglia [20]. To date, there have been no studies on the distribution and localization of GLP-1R in the TNC. For the first time, we investigated that GLP-1R was widely expressed in the TNC and was specifically localized to microglia and astrocytes using immunofluorescence staining. Our western blotting data showed that GLP-1R protein expression in the TNC was increased after recurrent NTG injection. However, there is a major limitation that our present study includes only male animals. We have indicated that male mice could also successfully establish NTG induced CM model [17, 18], it is well known that sex differences in migraine is mainly due to the role of estrogen [44, 45], thus, to avoid the influence of estrogen, we selected male mice for further study. Whether the distribution and activation of TNC GLP-1R in female animals are different remains to be further studied.

We have indicated that the number of microglia in the TNC is also increased in this study and our previous studies [17, 18]. However, we did not find a significant upregulation of astrocytes in the TNC in this NTG-induced CM model in our previous researches. Considering the consistency of the alterations in GLP-1R and microglia in the TNC, we predict that the main reason for the upregulation of GLP-1R is due to the increased number of microglia. Consistent with our data, the upregulation of GLP-1R in the spinal cord on activated microglia was also found after peripheral nerve injury [20]. However, data on the changes in GLP-1R levels in other CNS disorders accompanied by microglial activation are contradictory. For example, the expression of GLP-1R in Alzheimer’s disease, experimental autoimmune encephalomyelitis and depression [46–48] was found to be decreased in the brain. The differences in GLP-1R expression in activated microglia might be due to the different regions of the CNS and the various methods for activating microglia. Together, our data reveal that activating TNC microglia may increase the expression of GLP-1R in CM mice in response to chronic NTG injection.

Our behavioural results showed that chronically activating GLP-1R by means of consecutive i.p. injections of the selective agonist liraglutide attenuated NTG-induced basal tactile allodynia. However, blocking GLP-1R did not improve CM-related hypersensitivity. This finding suggests that liraglutide mainly produces prophylactic analgesic effects. Liraglutide, a GLP-1 analogue, is a new therapeutic drug for the treatment of diabetes. The drug can cross the blood-brain barrier (BBB) and activate GLP-1R in the CNS [49, 50], thus producing neuroprotective and anti-inflammatory effects [50–55]. Previous data demonstrated that activation of GLP-1R could alleviate neuropathic pain, cancer pain and diabetic neuropathy [20]. In the present study, we provide the first evidence that liraglutide prevented the development of CM by activating GLP-1R in the TNC. It is known that CM shares a common mechanism of central sensitization with chronic pain; thus, our data complement the impact of GLP-1R on chronic pain.

It is well known that GLP-1Rs themselves are inactive and needs to be activated or inhibited to exert their function. Therefore, to clarify whether the function of GLP-1R is activated or inhibited in CM, we detected the changes in the level of endogenous GLP-1, a natural agonist of GLP-1R, in the TNC in NTG-induced CM mice. We performed western blotting to examine the protein level of endogenous GLP-1 in the TNC during repeated NTG administration and also conducted double-staining of GLP-1 and iba-1 to further detect the distribution of GLP-1 in the TNC. The results showed that GLP-1 was co-localized with microglia in the TNC, recurrent NTG administration decreased the number of GLP-1-positive cells compared with that in the sham group (Fig. S1). These results suggested that GLP-1 expression was gradually decreased in CM, and also revealed that although the protein expression of GLP-1R was upregulated after NTG stimulation, the amount of GLP-1Rs that can be activated was decreased, so the activity of GLP-1R in the TNC in CM model was actually inhibited. This data supports our behavioural analysis that activating GLP-1R by its agonist liraglutide plays a major role in relieving CM-related hyperalgesia.

Tactile allodynia is a clinical characteristics of CM patients which manifests as abnormal skin pain of the head and limbs [56], as we measured above (periorbital and paw responses); this is the external manifestation of central sensitization in the pathogenesis of CM. To further confirm the effect of GLP-1R on the central sensitization of CM, we examined the expression of CGRP and c-fos in the TNC. CGRP is a specific neuropeptide in the trigeminal system that plays a critical role in peripheral and central sensitization [9, 57]. Studies have indicated that NTG administration can induce the upregulation of CGRP in the TNC, dura mater, and blood [57]. C-fos, an immediate early gene that is widely considered a marker of neuronal activity, is also involved in central sensitization [58]. Studies in different animal models of migraine (NTG, CSD and IS stimulation) demonstrate a significant upregulation of c-fos in laminae I and II of the TNC [59]. Our findings showed that activating GLP-1R by i.p. injection of liraglutide dramatically suppressed the upregulation of CGRP and c-fos in the superficial lamina of the TNC induced by NTG. This result, combined with our behavioural data, confirms that activation of GLP-1R in the TNC may inhibit the central sensitization of CM.

Accumulating evidence has revealed that changes in microglial functions (for example, morphological changes, process movement, and inflammation) are required for the development of chronic pain [60–62]. Our previous studies have indicated that microglial activation in the TNC contributes to the central sensitization of CM [17, 18]. Thus, we further explored whether GLP-1R is involved in CM by mediating microglial activation, including morphological changes and inflammation. In this study, chronic NTG injection induced an increase in microglial cell numbers and a significant reduction in microglial process length and inflammatory factor (IL-1β and TNF-α) release. Following liraglutide treatment, the upregulation of microglial cell number and proinflammatory factors was markedly decreased, and microglia exhibited much longer process lengths in the TNC. However, since the drugs were administered systemically in a simulated clinical manner, we cannot rule out the effect of GLP-1R in the peripheral system. Thus, we carried out experiments in BV-2 microglia to more accurately determine the effect of GLP-1R on microglial function. Consistent with the results in the CM animal model, GLP-1R is completely colocalized with BV-2 microglia, and activation of GLP-1R by liraglutide reduced Iba-1 protein expression and inflammatory factors. From these data, we conclude that GLP-1R is involved in regulating TNC microglial activation during NTG injection, and this function of GLP-1R might also be the mechanism for attenuating CM-related allodynia. Although our data *in vivo* and in *vitro* demonstrated that activating GLP-1R inhibited microglial activation, changes in microglia induced by liraglutide can be indirect as well as behavioral effects could be driven by other cells cause our treatments are delivered systemically. Because of the limit of this study, the potential role of GLP-1R in the TNC still needs to be further explored.

Studies have indicated that GLP-1R is involved in regulating cell proliferation and migration, neuronal activity, and inflammation through its downstream PI3K/Akt pathway [23, 42, 63]. Moreover, PI3K/Akt has been reported to be upregulated in migraine [64], but no study has provided a direct evidence of whether this pathway participates in the mechanism of CM. Our study confirmed that the PI3K/Akt pathway acts downstream of GLP-1R in the TNC in CM mice. In addition, we observed that the protein expression of PI3K and p-Akt in the TNC gradually increased in the CM model, and inhibiting the PI3K/Akt pathway by repeated administration of the selective antagonist LY294002 relieved NTG-induced basal tactile allodynia. These results provide the first evidence that inhibiting the PI3K/Akt pathway attenuates CM-associated allodynia; that is, the pathway participates in the central sensitization of CM. The involvement of GLP-1R in the pathogenesis of CM may also be realized by regulating the activity of this pathway.

## Conclusions

**Fig. 9:**
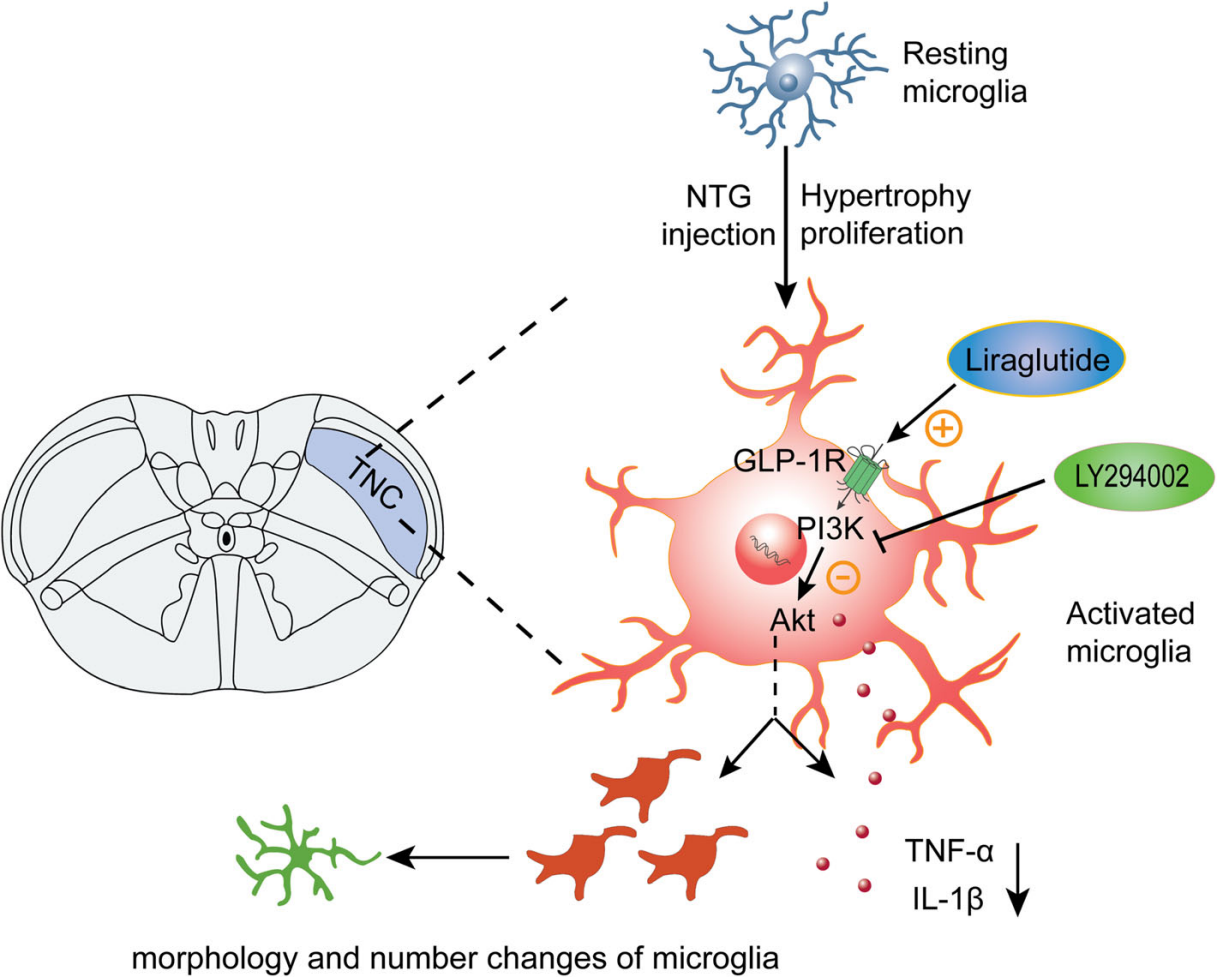
Schematic of the potential mechanisms of GLP-1R in the TNC in the CM animal model. After repeated NTG administration, microglia in the TNC are activated, and GLP-1R expressed on microglia is dramatically upregulated. Activation of GLP-1R inhibits the function of the downstream PI3K/Akt pathway, thereby inhibiting microglial cell proliferation, morphological changes (shortening of the processes), and inflammatory cytokine (IL-1β and TNF-α) production and ultimately alleviating the central sensitization of CM

Our study demonstrated that the expression of GLP-1R is increased in TNC microglia after NTG injection. Activation of GLP-1R with its selective agonist liraglutide alleviated CM-associated allodynia and reduced the expression of CGRP and c-fos in the TNC. We also found that liraglutide inhibited NTG-induced microglial morphological changes (process retraction) and proinflammatory factor (IL-1β and TNF-α) production in the TNC. These functions of GLP-1R in the CM model is mediated by its downstream PI3K/Akt pathway (Fig. 9). These findings provide new insights into the pathogenesis of CM and suggest that microglial GLP-1R in the TNC may be a new target for the treatment of CM. The GLP-1R agonist liraglutide, which is a commonly used hypoglycaemic drug in the clinic, might represent a new therapeutic approach for treating CM.

## Supplementary Information

**Additional file 1: Figure S1.** The expression and localization of GLP-1 in the TNC in the sham and NTG (9d) groups.

## Data Availability

The datasets used and analyzed in the present study are available from the corresponding author on reasonable request.

## Abbreviations

BBB: Blood-brain barrier
CGRP: Calcitonin gene-related peptide
CM: Chronic migraine
CNS: Central nervous system
CSD: Cortical spreading depression
DMEM: Dulbecco’s modified Eagle’s medium
FBS: Foetal bovine serum
GLP-1R: Glucagon-like peptide-1 receptor
IGF-1: Insulin-like growth factor-1
IS: Inflammatory soup
LPS: Lipopolysaccharide
NTG: Nitroglycerin
PI3K: Phosphoinositide 3-kinase
PKA: Protein kinase A
PMSF: Phenylmethylsulfonyl fluoride
RT: Room temperature
TNC: Trigeminal nucleus caudalis
