# Supplementary material for: Activation of microglial GLP-1R in the trigeminal nucleus caudalis suppresses central sensitization of chronic migraine after recurrent nitroglycerin stimulation
Source: J Headache Pain. 2021 Jul 29;22(1):86. doi: 10.1186/s10194-021-01302-x (PMC8323319; doi:10.1186/s10194-021-01302-x)
Supplement: Supplementary file 1 — Additional file 1: Figure S1. The expression and localization of GLP-1 in the TNC in the sham and NTG (9d) groups. [file 10194_2021_1302_MOESM1_ESM.docx]

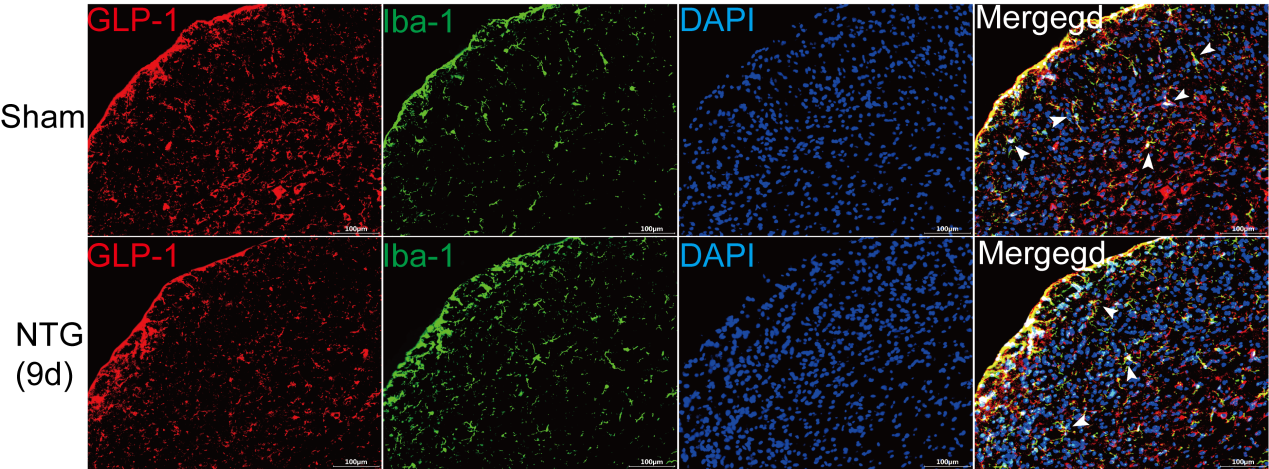


**Figure S1. The expression and localization of GLP-1 in the TNC.** Representative double-immunofluorescence images of GLP-1 (red) and iba-1 (green) show the expression of GLP-1 in sham and NTG (9d) groups, and suggest that GLP-1 is co-localized with microglia in the TNC. Arrowheads show double-labelled cells. Scale bar: 100 μm.
